# Site-specific N-glycosylation of HeLa cell glycoproteins

**DOI:** 10.1038/s41598-019-51428-x

**Published:** 2019-10-15

**Authors:** Lilla Turiák, Simon Sugár, András Ács, Gábor Tóth, Ágnes Gömöry, András Telekes, Károly Vékey, László Drahos

**Affiliations:** 1 MS Proteomics Research Group, Research Centre for Natural Sciences, Magyar tudósok körútja 2, H-1117 Budapest, Hungary; 2 Semmelweis University, Ph.D. School of Pharmaceutical Sciences, Üllői út 26, H-1085 Budapest, Hungary; 3 Budapest University of Technology and Economics, Faculty of Chemical Technology and Biotechnology, Műegyetem rakpart 3, H-1111 Budapest, Hungary; 4 Department of Oncology, St Lazarus County Hospital, Füleki út 54-56, H-3100 Salgótarján, Hungary

**Keywords:** Cervical cancer, Glycomics, Proteomics

## Abstract

We have characterized site-specific *N*-glycosylation of the HeLa cell line glycoproteins, using a complex workflow based on high and low energy tandem mass spectrometry of glycopeptides. The objective was to obtain highly reliable data on common glycoforms, so rigorous data evaluation was performed. The analysis revealed the presence of a high amount of bovine serum contaminants originating from the cell culture media – nearly 50% of all glycans were of bovine origin. Unaccounted, the presence of bovine serum components causes major bias in the human cellular glycosylation pattern; as is shown when literature results using released glycan analysis are compared. We have reliably identified 43 (human) glycoproteins, 69 *N*-glycosylation sites, and 178 glycoforms. HeLa glycoproteins were found to be highly (68.7%) fucosylated. A medium degree of sialylation was observed, on average 46.8% of possible sialylation sites were occupied. High-mannose sugars were expressed in large amounts, as expected in the case of a cancer cell line. Glycosylation in HeLa cells is highly variable. It is markedly different not only on various proteins but also at the different glycosylation sites of the same protein. Our method enabled the detailed characterization of site-specific *N*-glycosylation of several glycoproteins expressed in HeLa cell line.

## Introduction

HeLa is the most commonly analyzed immortalized cell line in biomedical research^1^. It is a human cervical adenocarcinoma cell line expressing over 10 000 proteins^2^. The tryptic digest of HeLa S3 cells is commercially available and is the most widely characterized mass spectrometry standard^2–5^ in proteomics. The mixture contains various post-translational modifications that have been studied as well^6–8^. The cell lysate has been used for method development purposes in case of phosphoprotein analysis^6,9^, stable isotope coded expression proteomics studies^10^ and glycopeptide enrichment^11^.

Although frequently used, there is only limited information available on the *N*-glycosylation of the HeLa glycoproteins. Understanding *N*-glycosylation patterns is important, as almost all cell surface proteins are glycosylated and it is assumed that without these glycoproteins the cells cannot survive^12^. It is not yet completely clear how the *N*-glycans influence the function or the fate of the *N*-glycoproteins. *N*-glycan residues may regulate receptor affinity due to influencing the folding, by altering the polarity and solubility of the ligand or the receptor, as well as binding to extra- or intracellular molecules which participate in signal transduction pathways^13^. It was suggested that glycans may protect enzymes on the cell surface from being degraded^14^. *N*-glycoproteins are synthesized within the cells, mainly in the endoplasmic reticulum and in the Golgi apparatus^15,16^.

*N*-glycosylation sites of HeLa cellular proteins are fairly well referred using hydrazide enrichment followed by nanoLC-MS/MS analysis^11^. Using this method 268 unique *N*-glycosylation sites corresponding to 106 glycoproteins have been identified so far^11^. In two recent articles, glycopeptides were enriched from the HeLa cell lysate^7,17^ followed by cleaving the sugars from the glycopeptides. Subsequent mass spectrometry analysis was used to locate the site of glycosylation but the attached sugar structures were not studied.

Cell surface *N*-glycome of HeLa cells has also been characterized. The living cells were incorporated into polyacrylamide gels, the *N*-glycans released and 2-AB labelled then separated using HILIC chromatography^18^. Applicability of filter aided *N*-glycan separation for released *N*-glycan analysis has also been demonstrated on HeLa cells^19^. The released *N*-glycans were permethylated and analyzed using MALDI-MS. The method was suitable to detect differences between *N*-glycans of treated and non-treated HeLa cells. It was recently shown that the activation of the XBP1 transcription factor can increase the tetra-antennary *N*-glycan population in HeLa cell membrane^20^. However, much less is known about intracellular *N*-glycoproteins^21^. Glycosylation is known to have a significant role in cancer, for example in the stability and intracellular trafficking of glucose transporter GLUT4^22^. Aberrant glycosylation is considered a hallmark of cancer since glycosylation is involved in malignant properties of cancer cells^23,24^.

The missing part in these studies is the information on the glycosylation of the individual proteins and on the specific glycosylation sites. Detailed characterization can be obtained by glycopeptide analysis, which yields protein and site-specific *N*-glycosylation patterns; i.e. identifying which glycoforms are present (and in which proportion) at individual glycosylation sites of various HeLa proteins. A further advantage of this approach, which will be highlighted in the present paper, is that it is highly tolerant to various impurities which may be present in the sample.

Our aim was to characterize the site-specific *N*-glycosylation profiles of the individual glycoproteins expressed in HeLa cells, analyzing tryptic glycopeptides in a commercially available HeLa cell digest. This information will extend the possible use of HeLa cells as proteomics and mass spectrometry standards.

## Results

Initial proteomics analysis of the commercial, widely used HeLa standard revealed that the sample contains a large amount of bovine serum proteins, most likely from fetal bovine serum (FBS), present in the cell culture media. We did not attempt quantitation, but approximately a third of the identified proteins in a nanoLC-MS/MS analysis of the HeLa digest were of bovine origin. In a given LC-MS run we have found 1129 human proteins, 716 bovine proteins, and 626 proteins, in which case it was not unequivocal if they were of human or of bovine origin (due to sequence similarities). In the course of glycosylation studies, both human and bovine glycopeptides were analyzed and the respective peak abundances were calculated. It was shown that approximately 50% of all glycopeptides derived from human and 50% from bovine proteins. The large amount of bovine proteins present in the commercial standard did complicate data analysis, but in most cases, it was possible to determine the glycosylation profiles of human proteins. Note, released glycan analysis (which is much more common than glycopeptide analysis) is unable to distinguish sugars derived from human or bovine proteins, and will, therefore, yield erroneous results.

**Figure 1:**
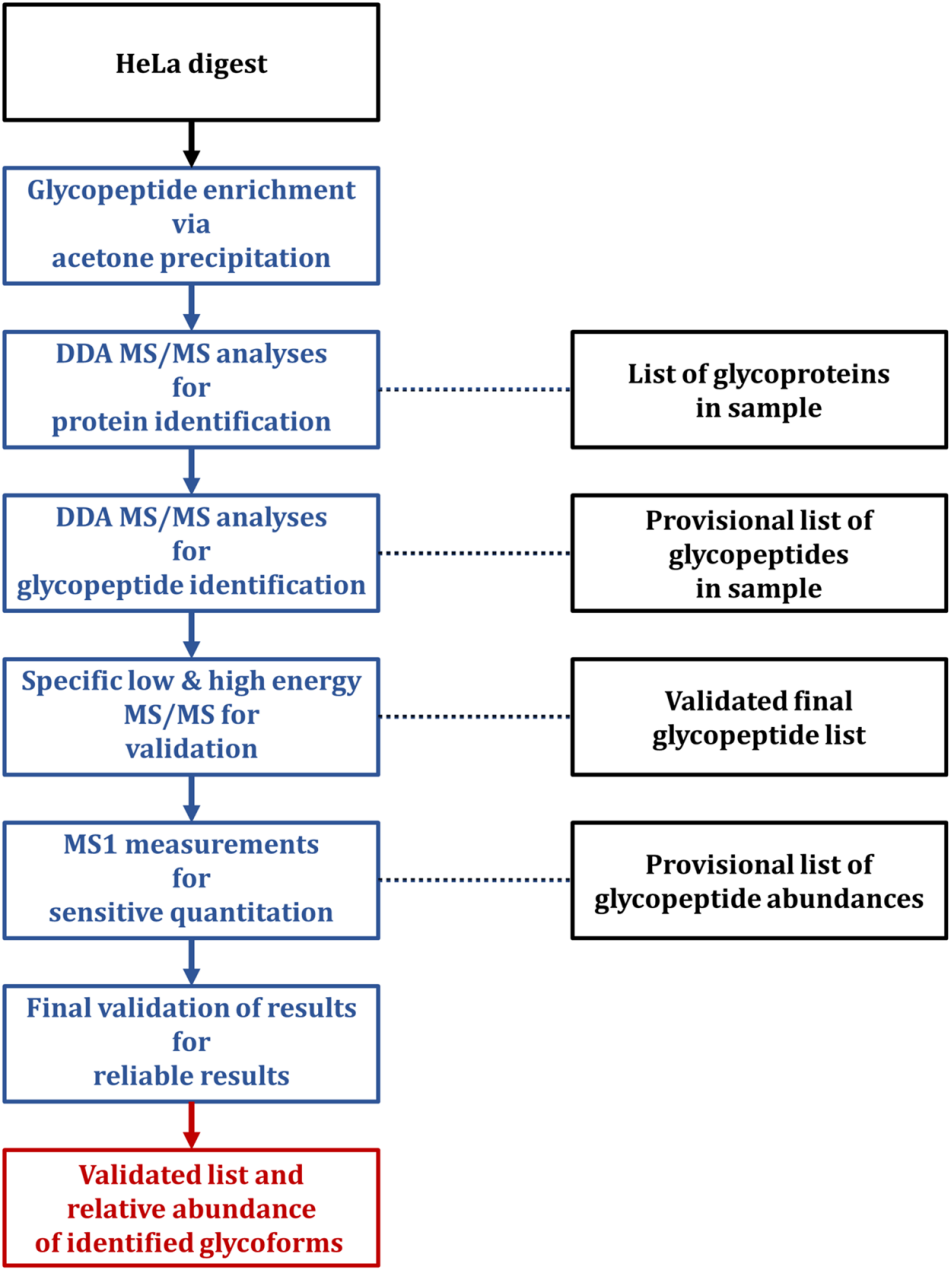
Overview of the workflow used in the present publication. Glycopeptides of the commercial HeLa digest were enriched using acetone precipitation. The glycopeptide enriched pellet fraction was subjected to DDA MS/MS analysis for glycoprotein and glycopeptide identification. Glycopeptides were validated using specific high and low energy MS/MS measurements. Quantitation was performed using single-stage mass spectrometry, and the results were validated using strict criteria. The validated site-specific glycosylation patterns are reported in Supplementary Table S2.

Characterizing site-specific *N*-glycosylation profiles of individual glycoproteins in a complex biological sample is challenging. There are various difficulties related to sample complexity, to the sensitivity of analysis and to structure identification. To overcome these issues, we have used a complex workflow to characterize site-specific *N*-glycosylation profiles of glycoproteins expressed in HeLa cells. The workflow is illustrated schematically in Fig. 1.

### Acetone precipitation

There are a large number of techniques used to enrich glycans, glycopeptides, and glycoproteins^25,26^. There are methods based on affinity to –OH groups (like boronic acid affinity separation/enrichment^27^), others on molecular recognition (like lectin affinity^28^), on chromatography (like HILIC^29^), and still others based on general chemical behavior (like strong cation exchange^30^) or on solubility (like acetone precipitation^31^). Detailed comparison of these methods is outside the scope of the present paper. Here we have performed a simple and cheap acetone solvent precipitation, which enriches glycopeptides present in a mixture, and is suitable to work with small sample amounts. The used method was based on a published protocol^31^, but we have optimized the amount of the organic solvent (acetone) and the pH of the aqueous solvent. Using the optimized protocol (see details in the Experimental part) we have precipitated most of the glycopeptides (pellet fraction), while most peptides remained in solution. This provides about 10-fold enrichment of the glycopeptides.

In order to determine peptide/glycopeptide amounts, data-dependent (DDA) LC-MS/MS studies were first performed to identify the major peptides and glycopeptides in the original sample, in the pellet, and in the supernatant. In a second step, single-stage LC-MS was performed to provide quantitation for these components, details will be described in the next section. The optimized enrichment protocol was tested for its efficiency and repeatability. Altogether five different pellets obtained at various times were analyzed. It was found that on average 91 ± 5% of the complex and 97 ± 1% of the high mannose *N*-glycopeptides were collected in the pellet. The pellet contained a variable amount of non-glycosylated peptides (5–15% of the total), likely due to incomplete removal of the supernatant. The site-specific *N*-glycosylation profiles of the glycopeptide-enriched pellet fraction were compared to that of the un-enriched HeLa digest. The correlation coefficient between glycopeptide abundances in the enriched and in the standard HeLa samples was 0.91, indicating that enrichment did not change the site-specific *N*-glycosylation profile significantly.

### Peptide and protein identification

Structure identification (sequencing) of peptides in the HeLa sample, in the pellet, and in the supernatant was performed using nanoUHPLC-MS/MS analysis in DDA mode. Peptide identification was performed under conventional conditions (see the Experimental part for details) and evaluated against Swissprot human and bovine databases. A known limitation of data-dependent analysis is that some compounds are not sampled in the LC-MS/MS run^5,32^. Furthermore, glycopeptides are among the low abundance peaks, so this problem is even more pronounced in the case of glycopeptide analysis. In order to obtain a more comprehensive result, the HeLa cell lysate was analyzed in triplicate and the results were combined (using the Byonic software^33^). The list of proteins (both human and bovine) present in the HeLa sample with a Byonic LogProb over 2.0 (p-value < 0.01) are listed in Supplementary Table S1.

### Identification of *N*-glycopeptides and glycosylation patterns

For glycopeptide identification, experimental conditions optimized for peptides are inadequate, as the sugar chains are easily lost, while the collision energy is insufficient to cleave the peptide backbone. To overcome these problems, a separate nanoUHPLC-MS/MS experiment was used to identify glycopeptides. A combined collision energy was used for MS/MS^34,35^, where the cycle time was divided between “high” and “low” energies. This yields glycopeptide MS/MS spectra containing information both on the peptide sequence and on the attached sugar chain. In order to decrease the likelihood of false-positive hits, the “glycopeptide-optimized” LC-MS/MS run was evaluated limiting the search only to those proteins, which had been identified during the proteomics analysis. Approximately two times more *N*-glycopeptides were detected in the pellet fraction, than in the un-enriched HeLa sample. In order to increase glycopeptide coverage, LC-MS/MS analysis was done in triplicate, and the results were combined. This way 176 peptide sequences were found to be *N*-glycosylated in the enriched HeLa sample; among these 108 were of human, 39 of bovine origin; while 29 might be either bovine or human (as the peptide sequences are identical). Altogether 61 different oligosaccharide chains were observed among the glycan parts of the detected *N*-glycopeptides. This resulted in a *provisional* list of *N*-glycopeptides identified in the sample.

Despite the large number of structures identified, far more glycopeptides are present in the sample, but partly due to the limited sampling rate of DDA analysis, partly to their low abundance, many remain unidentified. In order to improve detection of glycopeptides of low abundance, a library of possible glycopeptides was first constructed. This contained the 61 oligosaccharides identified in LC-MS/MS analysis as described above, supplemented with 12 more sugar sequences, assumed to be reasonable based on known biosynthesis pathways. The full glycan library, therefore, contained 73 different oligosaccharides. This was combined with the 176 peptide sequences which were found to be glycosylated; creating altogether 12848 possible *N*-glycopeptides.

In order to identify low abundance glycopeptides, we performed nanoLC runs with single-stage MS detection. This is a comprehensive analysis (unlike DDA used above) and provides higher sensitivity than MS/MS. The obtained MS spectra were searched against the glycopeptide library, considering both the accurate mass and the isotope pattern of the glycopeptide. Peak identification and label-free quantitation were performed by the in-house developed GlycoPattern software^36^. Quantification was based on the peak area of the extracted ion chromatogram of the identified components (abundances of the various charge states were summed together). In order to improve glycopeptide coverage, reliability and precision of analysis, we have performed nanoLC-MS analysis in five replicates of enriched HeLa samples. Approximately 60% of all the identified glycopeptides (and 76% of those over 0.3% abundance) were found in all 5 replicates. The correlation coefficient of glycopeptide abundances in the various runs was, on average 0.98. Only those *N*-glycopeptides were accepted, which were identified at least in two out of five replicate analyses. This resulted in an extensive, but unvalidated, glycopeptide library of 859 glycopeptides. The abundance of these glycopeptides was taken as the average abundance of the replicate analyses.

### Manual validation of the results

A major effort was directed to confirm the validity of the results using (i) high energy MS/MS for glycopeptide backbone sequence validation; (ii) limiting database to highly confident hits only; (iii) confirming peptide sequences after glycan release; (iv) checking the expected and found retention times; and (v) using a statistical check to eliminate false or questionable hits. Validation has reduced the number of glycopeptide backbone sequences from 176 to 108; the number of *N*-bound oligosaccharide structures from 73 to 32; and the number of *N*-glycopeptides from 859 to 284. Note, these include both human and bovine glycopeptides. Among the validated glycopeptides those of human origin (altogether 178) are shown in Supplementary Table S2. Details of this validation are discussed below in detail.

**Figure 2:**
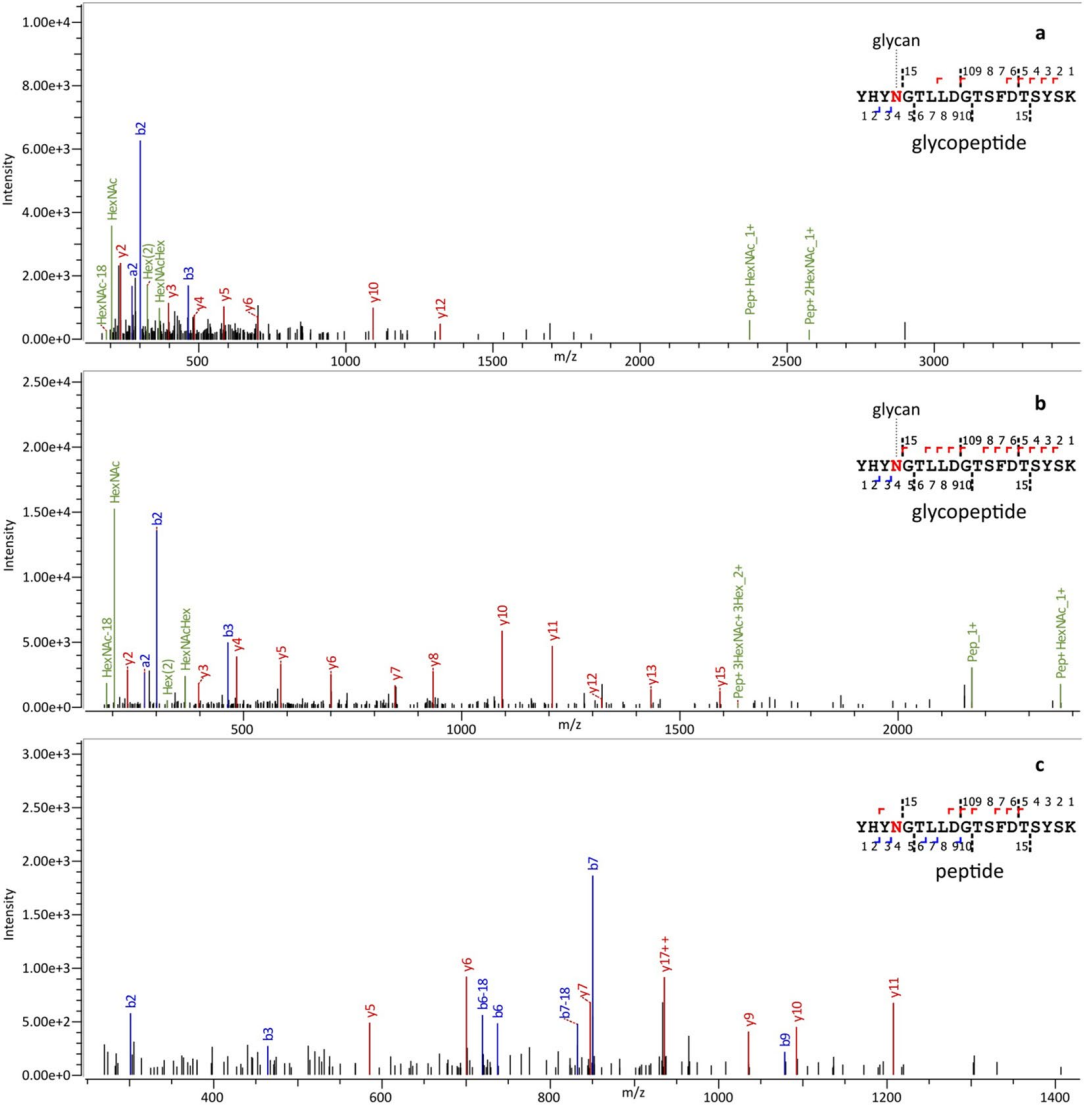
Example MS/MS spectra of glycopeptide YHYN*GTLLDGTSFDTSYSK-N2H8 (m/z 1291.1910) belonging to FKB10_HUMAN glycoprotein. (**a)** “glycopeptide-optimized” conditions using mixed energy, Byonic LogProb: 5.39, Byonic Score: 170 (**b**) 200% of the standard collision energy used in proteomics, Byonic LogProb: 8.38, Byonic Score: 454.9 (**c**) MS/MS of the corresponding peptide, obtained by digestion with PNGase F (standard collision energy). Byonic LogProb: 9.51, Byonic Score: 362.3.

Identification of the glycopeptide backbone using the “glycopeptide-optimized” experimental conditions is typically based on a few, low abundance backbone fragments. In order to improve sequence coverage, high energy MS/MS spectra were acquired (at 200% and 250% collisional energy compared that used in routine proteomics); using inclusion lists in separate LC-MS/MS runs. An example is shown in Fig. 2, which shows the MS/MS spectra of a glycopeptide (YHYN*GTLLDGTSFDTSYSK-N2H8) at the “glycopeptide-optimized” conditions (Fig. 2a) and at 200% of the standard collision energy used in proteomics (Fig. 2b); the latter shows a much higher sequence coverage. Note, combining different fragmentation energies for enhancing sequence coverage of glycopeptides has been demonstrated in several previous works as well^37–41^.

For each MS/MS spectra observed, the quality of glycopeptide identification is characterized by the Byonic LogProb and Byonic Score values. LogProb is the logarithmic p-value of the peptide-spectrum match (PSM) indicating statistical probability of a correct match; while the Score reflects the quality of the PSM. Among the 176 “*provisional*” glycopeptide backbones only those were considered reliable, where at least one MS/MS spectra were found with a LogProb value higher than 2.0 (p-value < 0.01) *and* a Byonic Score over 100. All other glycopeptide hits were excluded from the list of validated glycopeptides. All glycoproteins, which were identified by two peptide hits only (in the case of the standard proteomics study), were also considered unreliable and were excluded from further studies.

In a separate experiment, the enriched HeLa sample was digested by PNGase F, cleaving off the glycan groups. This sample was studied by LC-MS/MS under typical proteomics conditions, using DDA analysis. This analysis successfully identified many peptide sequences which, without using PNGase F, were glycopeptides. Such an example MS/MS spectrum is shown in Fig. 2c. This provided further confirmation for ca. 70% of the glycopeptide sequences identified, as discussed above.

Various glycoforms attached to the same peptide backbone elute at similar, but not identical retention times. For example, fucose substitution shortens the elution time by about 0.1 min; an added sialic acid increases it by about 1.5–2 minutes. A similar effect was discussed in detail before^41^. The retention times of all glycopeptides found as discussed above were manually checked, whether they confirm these expectations. Those, which were more than 1 min outside the expected retention time range, were considered false hits.

As the last step in the validation protocol, we have performed statistical checks to eliminate possible false hits. In the glycopeptide pool altogether 73 different oligosaccharide compositions were present. The 10 most common ones (excluding those of bovine origin) were defined as “common” oligosaccharide structures, and these are listed in Table 1. Those, which were found in less than 4 cases in the glycopeptide pool, were considered “rare” oligosaccharides. “Rare” glycoforms were accepted only in those cases, when (connected to the same peptide backbone) another, closely related “common” oligosaccharide was also found. For example, an oligosaccharide with and without fucose; or with one or two sialic acids were considered closely related. In an analogous manner those peptide sequences, which were found with one glycoform only, and this was not a common glycoform, were also excluded. The remaining 108 glycopeptide sequences, 32 *N*-bound oligosaccharide compositions, and 284 glycopeptides were considered validated as reliable hits, but some of these are derived from the bovine impurities present in the sample. Note, several glycopeptides eliminated in the validation protocol may actually be present, and using higher sensitivity many more could be found. However, here our objective was to create a database, which contains highly reliable results only.

**Table 1:** The top 10 most common oligosaccharide structures identified in the enriched HeLa cell lysate (N: N-acetylglucosamine, H: hexose, S: sialic acid, F: fucose).

Oligosaccharide Structure	Relative abundance	N-glycan Type	No. of glycoforms
N2H8	23.08	High-Mannose	32
N2H6	15.44	High-Mannose	23
N2H7	15.18	High-Mannose	18
N4H5S1F1	8.11	Complex	12
N4H5F1	5.18	Complex	11
N2H5	4.65	High-Mannose	6
N4H5S2F1	4.43	Complex	11
N4H5S2	4.41	Complex	5
N2H9	3.11	High-Mannose	11
N3H6	2.70	Hybrid	4

The commercial HeLa sample contained a massive amount of bovine proteins, likely from the FBS culture media. Among the validated glycopeptide hits, we have found 43 human glycoproteins with 69 glycosylation sites (glycopeptide sequences), 28 different oligosaccharide compositions, and altogether 178 glycopeptides. These comprise the validated human HeLa glycopeptide library and are listed, with relative abundances, in Supplementary Table S2. Major features of the 10 most abundant glycosylation sites in the HeLa cell line are shown in Table 2. There were 15 further glycoproteins (17 glycosylation sites, 44 glycopeptides), in which case the human and bovine peptide sequences were identical, and it was impossible to decide if they were human or bovine proteins. For completeness, these are listed in Supplementary Table S3. The corresponding relative abundances were normalized to the sum of all valid HeLa glycopeptides. The sample also contained 28 bovine serum proteins (altogether 62 glycopeptides). The total abundances of these bovine glycopeptides were approximately the same as those of human (HeLa) origin.

**Table 2:** The 10 most abundant, validated human glycosylation sites and their relative abundances in the enriched HeLa cell lysate.

Glycoprotein	Peptide backbone	Glycosylation site	Relative abundance	High-mannose %	Hybrid %	Complex %	Degree of sialylation %	Degree of fucosylation %
TFR1_HUMAN	QNNGAFN * ETLFR	727	8.9	100.0	0.0	0.0	—	—
CBPM_HUMAN	NFPDAFEYNN * VSR	164	8.5	92.1	0.0	7.9	0.0	100.0
LAMP1_HUMAN	GHTLTLN * FTR	103	7.6	69.6	9.6	20.7	53.8	77.8
FOLR1_HUMAN	NAccSTN * TSQEAHK	69	4.3	0.0	4.6	95.4	59.3	100.0
DAF_HUMAN	GSQWSDIEEFcN * R	95	4.0	0.0	0.0	100.0	91.7	8.4
LAMP1_HUMAN	LLNINPN * K	261	4.0	23.6	35.2	41.2	3.5	84.0
PLOD1_HUMAN	YIHQN * YTK	538	3.9	42.6	57.4	0.0	0.0	0.0
FOLR1_HUMAN	GWN * WTSGFNK	161	3.7	0.0	0.0	100.0	50.0	72.8
LAMP2_HUMAN	VQPFN * VTQGK	356	3.2	81.0	8.6	10,4	0.0	0.0
NICA_HUMAN	IYIPLN * K	45	2.5	13.3	52.0	34.7	44.5	100.0
**Distribution among all validated human glycoforms**				64.9	5.1	30.0	46.8	68.7

Glycosylation site is the position of the glycosylated asparagine in the glycoprotein’s amino acid sequence. Sum of the abundance of all glycoforms corresponding to the given site and the percentage of different *N*-glycan structure types are also given, together with the degree of sialylation (ratio of sialic acid and galactose residues) and degree of fucosylation (ratio of fucosylated glycoforms compared to the sum of complex and hybrid glycoforms).

### Structure analysis of the oligosaccharide part of glycopeptides

**Figure 3:**
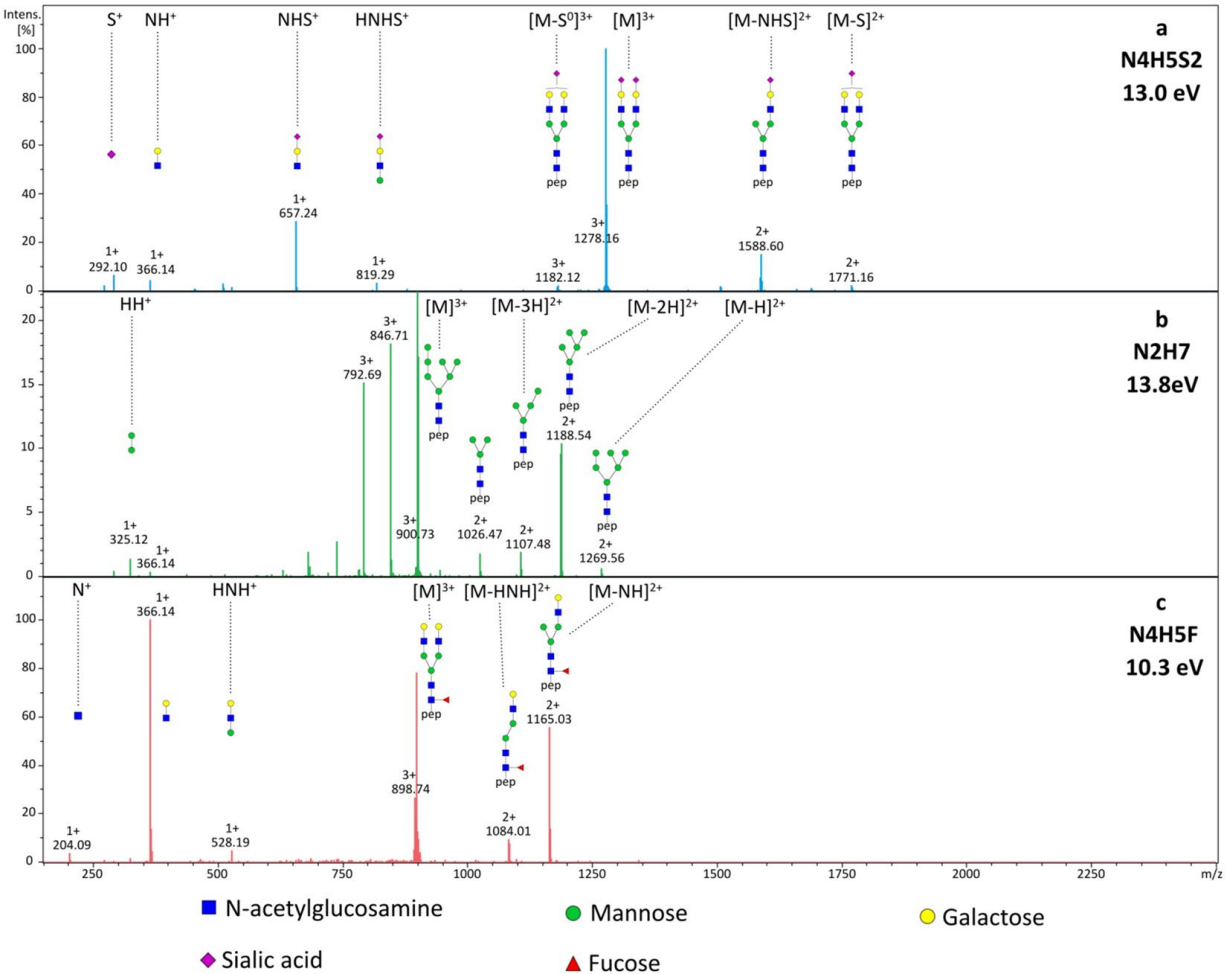
Low energy MS/MS spectra of selected human glycopeptides. (**a**) GSQWSDIEEFCNR –N4H5S2 glycopeptide (*m/z* 1278.1558) derived from DAF_HUMAN at 13 V (35% of the “standard” collision energy). (**b**) GHTLTLNFTR –N2H7 glycopeptide (*m/z* 900.7216) derived from LAMP1_HUMAN at 13.8 V (40% of the “standard” collision energy). Fragment intensities were enlarged by a factor of 5 for better visibility. (**c**) LLNINPNK –N4H5F glycopeptide (*m/z* 1347.5967) derived from LAMP1_HUMAN at 10.3 V (30% of the “standard” collision energy). (N: N-acetylhexosamine, H: hexose, S: sialic acid, F: fucose).

Automatic data evaluation procedures may identify glycan composition (monosaccharide content) but do not give information on the sugar structure. Experimental procedures typically use medium or high energy spectra, where often several consecutive fragmentation steps take place^41,42^. Glycopeptide identification is far better at low collision energy^41,42^ (when the precursor ion is the most abundant peak). Low energy spectra were collected for the most abundant glycopeptides and were manually evaluated, using procedures analogous to those published recently^42^. In all cases, the triply protonated precursor was selected for MS/MS using inclusion lists, and spectra were studied at 20, 30, 35 and 40% that of the collision energy typically used for proteomics. This allowed confident structural elucidation of the sugar side chains and provided further confirmation of the results shown in Table 2. Examples for low energy MS/MS spectra of a complex, a high mannose, and a complex, core-fucosylated glycopeptide identified in the HeLa mixture are shown in Fig. 3.

Figure 3a shows a low energy MS/MS spectrum of a triply protonated complex biantennary, bisialylated glycan (with N4H5S2 composition), attached to the GSQWSDIEEFCNR peptide, corresponding to DAF_HUMAN glycoprotein. At the high mass range doubly charged ions corresponding to the loss of a singly charged sialic acid (S^+^) or a singly charged antenna (NHS^+^) are observed (these are [M-S]^2+^ and [M-NHS]^2+^, respectively). In the low mass range, the respective singly charged S^+^, NHS^+^ fragments are detected, clearly identifying the structure of the antenna.

Figure 3b shows the low energy MS/MS spectrum of a triply protonated high mannose glycan (with N2H7 composition), attached to GHTLTLNFTR; corresponding to the LAMP1_HUMAN protein. At the high mass end, the most abundant peak is due to HH^+^ (dihexose) loss; and at the low mass end, there is a corresponding, relatively abundant, oxonium ion (HH^+^). This suggests that there is no H3 antenna (otherwise H3^+^ loss would be at least as abundant); so the structure is most likely -NNH(H)(H5) or -NNH(H2)(H4) but not -NNH(H3)(H3). At the high mass end, there are other fragments due to Hn^+^ losses; these are most likely due to sequential fragmentations. In the middle mass range, there is a series of neutral H (hexose) losses from the triply protonated molecule; which is a characteristic feature of all high mannose structures.

The third example (Fig. 3c) is a fucosylated glycopeptide, LLNINPNK-N4H5F. Recently we have demonstrated that low energy tandem MS/MS can be used to distinguish core and antenna fucosylation^42^. Core fucosylation is related to cancer with regards to tissue invasion^43^ and metastasis^44^, so this is an especially relevant issue for HeLa cellular proteins. Using low energy CID experiments fucose migration is minimized^42^. When fucose is on the antenna, loss of both fucosylated and non-fucosylated antenna are observed, leading to NH^+^ and NFH^+^ loss and the presence of corresponding oxonium ions. When fucose is on the core, only NH^+^ loss (and NH^+^ ion) is observed. In Fig. 3c the latter case is observed (i.e. there is no [M-NHF]+ or NHF+ ion in the spectrum), so in this case, fucose is on the core.

## Discussion

To estimate the relative abundance of individual glycoproteins, label-free quantitation was performed on the un-enriched HeLa cell lysate. Based on the MaxQuant results only 5 out of the top 100 most abundant HeLa proteins were *N*-glycosylated. Altogether ca. 3.5% of the human proteins are *N*-glycosylated. Using acetone precipitation altogether 43 (human) glycoproteins and 69 *N*-glycosylation sites were reliably identified. The major novelty of the present publication is the identification of protein- and site-specific glycosylation patterns of these glycoproteins (Supplementary Table S2). Among the identified glycoproteins site-specific glycosylation had previously been identified only for one protein (EGFR_HUMAN), unrelated to HeLa cells. The N^352^ site of EGFR_HUMAN was reported to be glycosylated, but the structure of the attached oligosaccharides was not resolved^45^. In our study, we have identified that N3H5S1F1, N2H8 and N2H7 were the major glycoforms at this site. Among the 69 glycosylation sites found in this study, in 31 cases this is the first experimental evidence, that the site is indeed glycosylated; previously only the consensus sequon was known (Supplementary Table S2). Major glycosylation features of the 10 most abundant glycosylation sites are shown in Table 2, details are given in Supplementary Table S2.

Among the 69 glycosylation sites found in HeLa, 52 contained high mannose, 12 hybrid, and 32 complex *N*-glycans. In most proteins either high mannose or complex glycans were predominant. In some cases, however, two or even all three structure types were present (see Tables 2 and S2 for details). Previous results also showed high expression of high mannose type sugars in the HeLa samples^18^. High mannose glycans were shown to be involved in protein folding^46^, and their overexpression at the cell surface may be characteristics of cancer cells^47^.

Among the high mannose glycans N2H8, N2H7, and N2H6 structures were the most abundant. High mannose structures contained, on average, 7.1 mannose units. However, this varied significantly among the various glycoproteins. There were sites, like N^356^ site of LAMP2, where the short chain N2H5 variant was most abundant. At other sites, like N^727^ site of TFR1, the large N2H8 oligosaccharide structure was predominant. Among complex glycoforms, biantennary structures were most abundant (77%). There were many tri-antennary glycoforms, but with lower abundance (22%), and a few tetra-antennary structures (1%) were also found. Individual proteins showed a large diversity in this respect as well: there were only bi-antennary structures present on N^38^ site of CBMP; while in the case of the N^161^ site of FOLR1 bi- and tri-antennary structures were present in similar abundance.

**Figure 4:**
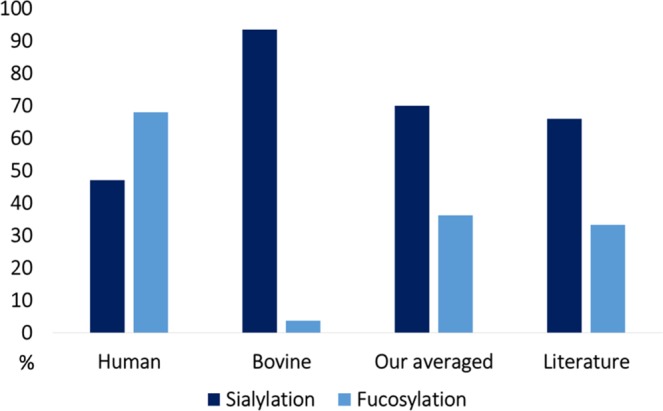
Degree of sialylation and fucosylation of human glycoproteins and bovine glycoprotein impurities. Values of human glycoproteins are different from those determined for the bovine glycoprotein impurities in the commercial HeLa sample. The abundance-weighted average of these is similar to that calculated from data in the literature based on released glycan analysis^48^.

HeLa glycoproteins are characterized by a high degree of fucosylation, on average 68.7% of hybrid and complex glycans were found fucosylated (Fig. 4). Using our recently developed low energy CID strategy^42^, we have identified that most of these were core fucosylation. One such example is shown in Fig. 3c. There is a large degree of variability in fucosylation among the various HeLa glycoproteins. For example, at the N^69^ site of FOLR1 only fucosylated glycoforms were found. On the other hand, the degree of fucosylation was only 8% in the case of the N^95^ site of DAF protein. Note that the bovine glycoprotein impurities present in HeLa were typically not fucosylated, their average degree of fucosylation was only 4% (Fig. 4).

The degree of sialylation is also an often studied aspect of complex N-glycans. The degree of sialylation (the percentage that the antennae are end-capped with sialic acid) is 46.8% in HeLa proteins on average (Fig. 4). This also shows a significant diversity among the various glycoproteins. The N^95^ site of DAF is nearly completely sialylated (92%), while site N^261^ of LAMP1 shows almost no sialylation (3.5%). Most other sites falling in between these two extremes. Bovine glycoprotein impurities, in contrast, were nearly completely sialylated (94% on average, Fig. 4).

Many HeLa proteins were glycosylated at several sites. Glycopeptide-based glycan analysis has the advantage of identifying these separately. For example, Table 2 shows two glycosylation sites (N^69^ and N^161^) for FOLR1. Site N^69^ has predominantly biantennary glycoforms, while site N^161^ shows bi- and triantennary structures in similar amounts; while no high mannose type glycans were observed at these sites (Supplementary Table S2). The degree of fucosylation and sialylation is similar at both sites. Supplementary Table S2 shows another protein (LAMP1) with 3 glycosylation sites. In this case, the glycosylation pattern at the three sites is completely different: site N^249^ contains bi-antennary glycans, site N^103^ contains predominantly high mannose glycans, while at site N^261^ high mannose, complex and hybrid structures are present in similar amounts (Supplementary Table S2).

The results discussed above show that glycosylation in HeLa and in bovine serum (impurity) is markedly different. By performing analysis based on glycopeptides we were capable of distinguishing most human and bovine proteins (based on the amino acid sequence).This is a clear advantage since, released glycan analysis is only able to give a weighted average of the glycan profile of the two species. In fact, Fig. 4 shows that the weighted average in the present study and results of a previous HeLa glycosylation analysis based on released glycan analysis^48^ show similar glycosylation features. This is a clear indication that previous results on HeLa glycosylation, based on released glycans, are misleading.

Our results demonstrate the highly variable nature of glycosylation in case of HeLa glycoproteins. Various glycoproteins and different glycosylation sites of the same glycoprotein are characterized by different glycosylation features. In the present paper, we list the major glycopeptides identified in HeLa. This may help to establish the HeLa cell line as a generally useful standard not only for proteomics but also for glycosylation studies. Furthermore, this may also be a starting point for the analysis of cancer-induced changes in glycosylation, and improve our understanding of the complex roles of individual glycoproteins.

We would also like to stress that the commercial HeLa cell line contains a high amount of bovine (glyco)proteins, likely from the cell culture media. Because of this identifying human glycosylation features is only possible when the analysis is based on (glyco)peptides, while released glycan analysis is misleading.

## Methods

### Chemicals, reagents and analytical standards

All chemicals used were HPLC-MS grade. Acetonitrile, Water, Acetone, Formic acid, and Ammonium-bicarbonate were purchased from Merck (Darmstadt, Germany). Trifluoroacetic acid, Dithiothreitol, Iodoacetamide, and HeLa protein digest standard were obtained from Thermo Scientific (Waltham, MA, USA). Methanol was purchased from VWR Chemicals (Radnore, PA, USA), RapiGest surfactant was obtained from Waters (Milford, MA, USA).

### Sample preparation

1 μL aliquot of the Thermo Scientific Pierce HeLa Protein Digest Standard (1 µg/μL) was dissolved in 15 μL water containing 1% formic acid, then 150 μL ice-cold acetone was added. The solution was stored at −20 °C overnight resulting in the formation of a pellet enriched in glycopeptides. Next day the sample was centrifuged at 12 000 g for 10 minutes. The supernatant was removed by careful pipetting, and the precipitate was dried in a vacuum centrifuge, then re-dissolved in 10 μL injection solvent (98% water, 2% acetonitrile, 0.1% formic acid). As for PNGase F deglycosylation, enriched glycopeptides (pellet fraction) were dissolved in 25 mM ammonium bicarbonate buffer (pH 8.5) in 100 ng/µL concentration. For de-glycosylation we treated the solution with 0.5 U PNGaseF enzyme/1 µg glycopeptide for 16 h at 37 °C. The reaction was stopped by heating the solution to 95 °C for 5 minutes.

The pellet and the supernatant fractions along with the untreated HeLa digest were studied by nanoLC-MS/MS and nanoLC-MS (see below).

### nanoUHPLC-MS(MS) analysis

Samples were analyzed using a Maxis II QTOF instrument (Bruker Daltonik GmbH, Bremen, Germany) equipped with CaptiveSpray nanoBooster ionsource coupled to a Dionex UltiMate 3000 RSLCnano system (Sunnyvale, CA, USA). Peptides were separated on an Acquity M-Class BEH130 C18 analytical column (1.7 μm, 75 μm × 250 mm Waters, Milford, MA) using gradient elution (4–50% linear gradient of eluent B in 90 min) following trapping on an Acclaim PepMap100 C18 (5 μm, 100 μm × 20 mm, Thermo Fisher Scientific, Waltham, MA) trap column. Solvent A consisted of water +0.1% formic acid, while Solvent B was acetonitrile +0.1% formic acid. For standard proteomics DDA measurements, the cycle time was set at 2.5 sec in the 700–2000 m/z range, preferred charges states were set between +2 and +5. MS spectra were acquired at 3 Hz, while MS/MS spectra at 4 or 16 Hz depending on the intensity of the precursor. In the case of the “glycopeptide-optimized” method mixed energy spectra were collected at 100% collision energy for 80% of the cycle time and 50% collision energy for 20% of the cycle time. For label-free quantitation, MS spectra were recorded over the mass range of *m/z* 300–3000 at 1 Hz. In the case of high and low energy measurements, the CID was performed on selected triply charged glycopeptide precursors using an inclusion list. The CID was performed at 4 Hz for abundant precursors and at 1 Hz for low abundant ones at various collision energies. First, the “standard” collision energy for precursor signals was determined following the manufacturer’s recommendations for peptides based on the isolation m/z, isolation mass range width and charge state of the ion. In the energy dependent measurements, the collision energy was set to a given percentage of this energy (in the 30–200% range). Following each run raw data were recalibrated using the Compass DataAnalysis software 4.3 (Bruker Daltonics, Bremen, Germany).

 Mass spectrometry data have been deposited to the ProteomeXchange Consortium via the PRIDE^49^ partner repository with the dataset identifier PXD013930.

### Qualitative and quantitative data analysis

Quantitative analysis of proteins from the MS/MS data was performed using MaxQuant software version 1.5.3.30^50^. Qualitative analysis of peptides and *N*-glycopeptides from the MS/MS data was done using Byonic software version 2.15.7^33^ (Protein Metrics Inc., Cupertino, CA, USA). LFQ analysis of glycopeptides and peptides from the MS1 data was done by using our in-house developed GlycoPattern software^41^. GlycoPattern requires a peptide and a glycopeptide backbone list with retention times (RT) derived from the Byonic search, and a summarized glycan list derived from the Byonic search, manual spectra evaluation, and glycopeptide synthesis pathways. For the additional manual spectra evaluation, multiple collision energies were used, corresponding to the different fragmentation properties of distinct glycoforms. Components were identified based on their charge state, m/z and RT values, and isotope distribution. Quantification was done by calculating the peak area of the Gaussian peaks fitted over the extracted ion chromatogram of the identified components. Results were then verified by performing random checks.

### Equipment and settings

Figure 1 was prepared using Microsoft PowerPoint and imported into Inkscape 0.92 software to create a vector graphic. Spectra in Fig. 2 were exported from Byonic software version 2.15.7 (Protein Metrics Inc., Cupertino, CA, USA) using default settings and further edited using Inkscape 0.92 software as follows. First, the label on top of the spectrum was removed containing the sequence of the peptide, charge state, scan number and scan time. Next, font sizes in the peptide sequence explaining fragmentation were increased and labels glycan, glycopeptide or peptide were added. Spectra in Fig. 3 were exported from Compass DataAnalysis software 4.3 (Bruker Daltonics, Bremen, Germany) and further edited using Inkscape 0.92 software as follows. First the label in the upper right corner was removed, then sugar structures explaining fragmentation of the glycopeptide were added, as well as the abbreviation of the N-glycan and the fragmentation energy. Figure 4 was prepared using Microsoft Excel and imported into Inkscape 0.92 software to create a vector graphic.

## Supplementary information

Supplementary Information

Supplementary Table S-1

Supplementary Table S-2

Supplementary Table S-3
