# Supplementary material for: Site-specific N-glycosylation of HeLa cell glycoproteins
Source: Sci Rep. 2019 Oct 15;9:14822. doi: 10.1038/s41598-019-51428-x (PMC6794373; doi:10.1038/s41598-019-51428-x)
Supplement: Supplementary file 1 — Supplementary Information [file 41598_2019_51428_MOESM1_ESM.pdf]

Supplementary Information for

Site-specific N-glycosylation of HeLa cell glycoproteins

Lilla Turiák, Simon Sugár, András Ács, Gábor Tóth, Ágnes Gömöry, András Telekes, Károly Vékey and László Drahos

Supplementary Table 1: The list of proteins (both human and bovine) identified in the HeLa cell lysate using Byonic software. Only proteins identified with a LogProb over 2.0 (p-value <0.01) are listed.

Supplementary Table 2: Validated human glycosylation sites, structure of glycans and their relative abundances in the enriched HeLa cell lysate. Glycosylation site is the position of the glycosylated asparagine in the glycoprotein's amino acid sequence. Sum of the abundance of all glycoforms corresponding to the given site and the percentage of different *N*-glycan structure types are also given, together with the degree of sialylation (ratio of sialic acid and galactose residues) and degree of fucosylation (ratio of fucosylated glycoforms compared to the sum of complex and hybrid glycoforms).

Supplementary Table 3: Human or bovine (human and bovine glycopeptide sequences are identical) glycosylation sites, structure of glycans and their relative abundances in the enriched HeLa cell lysate. Values are normalized to the sum of all identified and validated human HeLa glycopeptides.
